# The anthropogenic effect of land use on population genetics of *Malcus inconspicuus*

**DOI:** 10.1111/eva.13512

**Published:** 2022-12-05

**Authors:** Shujing Wang, Yanfei Li, Jiayue Zhou, Kun Jiang, Juhong Chen, Zhen Ye, Huaijun Xue, Wenjun Bu

**Affiliations:** ^1^ Institute of Entomology, College of Life Sciences Nankai University Tianjin China

**Keywords:** cropland, evolutionary history, habitat loss, *Malcus inconspicuus*, mountain environment

## Abstract

Since the beginning of the Holocene era, human activities have seriously impacted animal habitats and vegetative environments. Species that are dependent on natural habitats or with narrow niches might be more severely affected by habitat changes. *Malcus inconspicuus* is distributed in subtropical China and highly dependent on the mountain environment. Our study investigated the role of the mountainous landscape in the historical evolution of *M. inconspicuus* and the impact of Holocene human activities on it. A phylogeographical approach was implemented with integrative datasets including double‐digest restriction site‐associated DNA (ddRAD), mitochondrial data, and distribution data. Three obvious clades and an east–west phylogeographical pattern were found in subtropical China. Mountainous landscape has “multifaceted” effects on the evolutionary history of *M. inconspicuus*, it has contributed to population differentiation, provided glacial refuges, and provided population expansion corridors during the postglacial period. The effective population size (*Ne*) of *M. inconspicuus* showed a sharp decline during the Holocene era, which revealed a significantly negative correlation with the development of cropland in a hilly area at the same time and space. It supported that the species which are highly dependent on natural habitats might undergo greater impact when the habitat was damaged by agricultural activities and we should pay more attention to them, especially in the land development of their distribution areas.

## 1 INTRODUCTION

Since the beginning of the Holocene era, also termed the human era, human activities have had a great impact on the Earth's ecosystems, and today, most of terrestrial nature and global biodiversity have been reshaped by anthropogenic activity (Bernardo‐Madrid et al., 2019; Boivina et al., 2016; Ellis et al., 2021; Kemp et al., 2020). Intensified human activity, particularly habitat disturbance and overexploitation, is considered to be the direct cause of genetic decline, population extirpation, and even species extinction (Johnson et al., 2017). The excessive development of agriculture has left the greatest impact on phytophagous animals, resulting in the loss of natural habitats and changes in the composition of local vegetation (Raven & Wagner, 2021).

Many in‐depth studies have analyzed and revealed the causes of the current survival state of some remarkable species (Curry et al., 2021; Dong et al., 2021; Nater et al., 2015; Wei et al., 2014) from the aspects of evolutionary history and current human activities. For example, climatic fluctuations in the Quaternary glacial period have caused genetic bottlenecks in giant pandas (Wei et al., 2014), red pandas (Hu et al., 2011), and Yunnan snub‐nosed monkeys (Liu et al., 2009). After entering the Holocene era, human interference has led to a further decline in their population. Therefore, their current endangerment status is likely attributable to regional environmental pressure, including historical climate turbulence and recent human disturbance. Thus, the information on population evolutionary history and the genetic diversity level of the species found in specific areas is necessary to understand their current distribution pattern and survival state.

Insects, as an important group of invertebrates, have the highest species richness and constitute the main body of terrestrial diversity (Stork, 2018). It is worth noting that, about half of all insect species are phytophagous, which makes them tightly associated with their host plants found in their habitats (Basset & Lamarre, 2019). Habitat loss is probably the most important threat faced by temperate and tropical insects (Newbold et al., 2018), and agricultural expansion has fueled this habitat loss (Sánchez‐Bayo & Wyckhuys, 2019). Among them, species that are dependent on natural habitats or with narrow niches (such as dependence on particular host plants, ecological niches, or restricted habitats) might be more severely affected by habitat changes. For many bumblebees and wild bees (Williams & Osborne, 2009), the change in land use (including loss of floral resources, nesting, and hibernation sites) seemed to be the decisive factor in their reduction. For some specialist ground beetles (Brooks et al., 2012), the loss of hedgerows and trees in the habitat likely triggered the decline in their numbers. Moreover, as the host plants of overwintering larvae reduces, the number of moths also decreases gradually (Fox, 2013; Mattila et al., 2006; Merckx et al., 2009; Pocock & Jennings, 2008). Therefore, the species with high habitat dependence will be more sensitive to the impact of human activities on their population dynamics.

*Malcus inconspicuus* is mainly distributed in the subtropical region of China, where the mountain area is vast and the trend of mountains is diverse (Wang & Bu, 2020). Mountain areas are considered evolutionary cradles of speciation (García‐Rodríguez et al., 2021; Li et al., 2021), and also provided both refuge and dispersal corridors for species during periods of large climate change. Through field investigation, we found that *M. inconspicuus* is an oligotrophic phytophagous insect and primarily lived in hilly landscapes (Figures S1–S12), including Gulong mountain (on the eastern edge of Yunnan Guizhou Plateau), Nanling mountain, Luoxiao mountain, and Wuyi mountain, which are highly dependent on the mountain environment. Therefore, the change in the mountainous environment in subtropical China might have an impact on their survival status. Moreover, previous research by the authors on the phylogeography of species in *Malcus* Stål, 1859, revealed that the population historical dynamics of *M. inconspicuus* may be disturbed by human activities.

In this study, we integrated ddRAD data, mitochondrial data, and distribution data of *M. inconspicuus* to reveal current phylogeographic distribution patterns and reconstruct the historical evolutionary process of populations driven by historical climate fluctuations. Meanwhile, we combined the population's historical dynamics with various human disturbance factors to explore the impact of human disturbance on the species.

## 2 METHODS

### 2.1 Sampling, DNA extraction, ddRAD library preparation, and mitochondrial reference preparation

**FIGURE 1:**
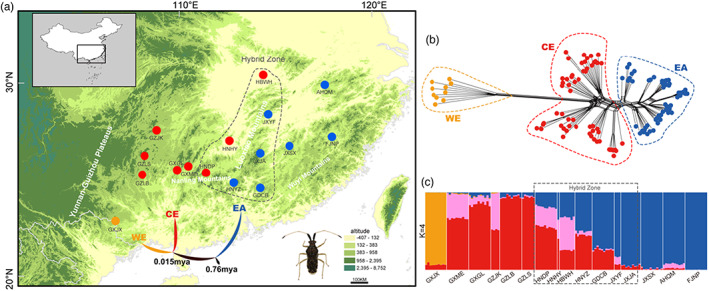
Phylogeographical analysis results based on ddRAD data of *Malcus inconspicuus* in subtropical China. (a) The geographical location of sampling; different colored dots represent three clades marked in the panel (orange—WE, red—CE, and blue—EA); the tree below the sampling dots represents the topological relationships and the historical divergent time based on the best‐fit demographic model; the morphological photograph is next to the tree. (b) Phylogenetic topology based on ddRAD data by neighbor‐net method. (c) Structure result with best *K* value.

A total of 128 samples across 16 populations of *M. inconspicuus* from China were used in this study (Figure 1, Table 1). All samples were maintained in 100% alcohol at −20°C in refrigerators. Voucher specimens were taken from each population and stored at the Institute of Entomology, College of Life Sciences, Nankai University, China (NKU). DNA was extracted from muscle tissue using a Universal Genomic DNA Kit (CWBIO). All ddRAD libraries were prepared following Peterson's protocol (Peterson et al., 2012). DNA genomics was double digested with *Eco*RI and *Msp*I restriction enzymes (New England Biolabs) and ligated with Illumina adaptors and unique 5 bp barcodes on both ends. Ligation products were pooled and ran through a Pippin Prep (Sage Science) to select fragments from 250 bp to 600 bp, followed by the amplification of size‐selected pools with eight cycles using PCR with Illumina indexed primers. The product of each step (enzyme digested, ligation, and amplified) was purified using AMPure XP magnetic beads (Beckman Coulter Inc.) before proceeding to the next step. Finally, paired‐end 150 bp reads were generated on an Illumina HiSeqX10 platform at the Novogene Sequencing Center & CAP and ISO Lab. In addition, one genomic DNA sample was selected to generate a reference of the mitogenome of *M. inconspicuus*. Sequencing was performed by Novogene with an insert size of 250 bp and a pair‐end 150 bp sequencing strategy on the Illumina platform. The mitogenome was assembled in MitoZ (Meng et al., 2019) and IDBA master (Peng et al., 2012) methods for assembly and mutual verification. The Mitos web server (http://mitos.bioinf.uni‐leipzig.de/idex.py/) (Bernt et al., 2013) was used to identify the boundaries of tRNA genes and their secondary structures using the invertebrate mitochondrial genetic code. The start and stop codons of protein‐coding genes (PCGs) were determined by the ORF Finder using invertebrate mitochondrial genetic codes on the NCBI website (https://www.ncbi.nlm.nih.gov/orffinder/). Two rRNA boundaries were determined by comparison with homologous regions of the other published *Malcus* mitogenomes in GenBank. Finally, the 13 new mitochondrial PCGs for *M. inconspicuus* were obtained as a reference.

**TABLE 1:** Nucleotide polymorphisms in each geographical population of *Malcus inconspicuus* for ddRAD datasets.

Clade	Population ID	*N*	Longitude	Latitude	Locality	*π*	*H*_O_	*H*_E_
EA	AHQM	10	117.57	30.05	Chiling village, Qimen County, Anhui Province, China	0.0397	0.2685	0.2897
	FJNP	10	117.95	27.36	JuKou Town, Jianyang District, Nanping City, Fujian Province, China	0.0286	0.3019	0.3143
	GDCB	10	114.23	24.70	Chebaling Nature Reserve, Guangdong Province	0.0550	0.2496	0.2755
	HNYZ	8	112.84	24.96	Xiaohuang Jialong village, Yizhang County, Hunan Province, China	0.0571	0.2732	0.3041
	JXJA	9	114.21	26.51	Lizhou village, Ji'an City, Jiangxi Province, China	0.0460	0.2824	0.2978
	JXSX	10	115.74	26.89	Shuangling bridge, Shangxi Township, Yongfeng County, Jiangxi Province, China	0.0447	0.2512	0.2750
	JXYF	3	114.62	28.53	Dianzibei village, Yifeng County, Jiangxi Province, China	0.0428	0.4317	0.4597
CE	GXGL	10	109.91	25.63	Huaping village, Longsheng County, Guilin City, Guangxi Province, China	0.0714	0.2412	0.2705
	GXME	10	110.47	25.83	Maoer Mountain, Guangxi Province, China	0.0693	0.2276	0.2582
	GZJK	4	108.84	27.70	Jiangkou County, Guizhou Province, China	0.0539	0.3773	0.4100
	GZLB	10	108.07	25.39	Maolan National Reserve, Libo City, Guizhou Province, China	0.0678	0.2496	0.2833
	GZLS	6	108.20	26.38	Leigong Mountain, Leishan County, Guizhou Province, China	0.0672	0.2913	0.3497
	HBWH	7	114.36	30.55	Wuhan University, Wuhan City, Hubei Province, China	0.0447	0.3303	0.3504
	HNDP	10	111.40	25.49	Yueyan forest farm, Dao County, Hunan Province, China	0.0643	0.2387	0.2665
	HNHY	1	112.61	27.15	Baishifeng village, Hengyang City, Hunan Province, China	0.0509	–	–
WE	GXJX	10	106.68	23.00	Gulong mountain canyon, Jingxi City, Guangxi Province, China	0.1001	0.2464	0.2945

Abbreviations: *N*, sample size; *π*, nucleotide diversity; *H*_O_, observed heterozygosity; *H*_E_, expected heterozygosity.

### 2.2 Bioinformatics

Processing of ddRAD‐seq data: The raw Illumina reads were processed with the “API: ipyrad assembly workflow” (Eaton & Overcast, 2020). First, we separated each individual into independent read files. Then, we confirmed the number of reads per individual and performed subsequent analysis (parameter set: the filter for the adapters was set to 2, the clustering threshold for de novo assembly was set to 0.85, and all other parameters were set at default values). Finally, an SNP_80 single nucleotide polymorphism (SNP) dataset (SNP_80 dataset) was generated, and each locus was required to be present in at least 80% of individuals (i.e., a maximum of 20% missing samples per locus). To avoid linkage across sites within the same locus, one random SNP was sampled from each locus and the USNP_80 dataset was finally generated for downstream analyses.

Mitochondrial data mining: Separated read files for each individual was mapped in Geneious Primer 2020.0.5 (https://www.geneious.com/), respectively, against each mitochondrial protein‐coding gene (PCG) of *M. inconspicuus*. We selected medium–low sensitivity as the mapping parameter because it is suitable for large numbers of high‐throughput sequencing reads. The consensus threshold was set to the highest quality as recommended by the software manufacturer. Consensus mapping required a minimum depth of three reads, the gaps and ambiguity codes were all replaced by Ns. The consensus sequences of each individual were trimmed based on the reference. Each PCG‐mapped dataset was aligned in mafft‐7.037 (Katoh & Standley, 2013). Finally, a concatenated 13 aligned PCG‐rad matric (PCG‐rad) was obtained.

### 2.3 Genetic polymorphism, phylogenetic analyses, and population genetic structure and differentiation

The genetic diversity was measured based on the SNP_80 dataset (containing 393,789 SNPs). The observed heterozygosity (*H*_O_) and the expected heterozygosity (*H*_E_) were calculated using Arlequin 3.5 (Excoffier & Lischer, 2010). The nucleotide diversity (*π*) was calculated in VCFtools (Danecek et al., 2011) and its correlation with longitude was graphically represented using the R package ggplot2 (Wickham, 2009). Phylogenetic analyses based on ddRAD and mitochondrial data adopted the neighbor‐net method and were implemented in SplitsTree v4.14.5 (Huson & Bryant, 2006). Population genetic structure based on USNP_80 was estimated using two methods: Bayesian clustering and DAPC. Bayesian clustering was performed in Structure 2.3.4 (Falush et al., 2003). We ran Structure 10 times for each *K* value from 2 through 16, to determine the optimal number of groups (*K*). Each run was performed for 200,000 Markov Chain Monte Carlo generations with a burn‐in period of 100,000 generations. Then, we used the Structure Harvester (http://taylor0.biology.ucla.edu/structureHarvester/) to find the best *K* value, combining 10 runs into one output in CLUMPP v1.1.2 (Jakobsson & Rosenberg, 2007) and graphically represented in distruct v1.1 (Rosenberg, 2004). DAPC was represented using the R package adegenet (Jombart, 2008). We used the three groups (EA, CE, and WE) based on the results of Bayesian clustering analysis and phylogenetic analysis as prior. The optimal number of PCs was determined using the xvalDapc() command. Bayesian analysis of population structure based on mitochondrial data was represented in BAPS 6.0 (Cheng et al., 2013). The pairwise *F*_ST_ values among sampling localities were estimated using R package hierfstat (Goudet, 2005) and graphically represented using the R package ggplot2 (Wickham, 2009).

### 2.4 Demographic history model testing and populations’ historical demographic reconstruction

In order to explore the specific evolutionary history of *M. inconspicuus*, we used Fastsimcoal v2.6.1 (Excoffier et al., 2013) to simulate the dynamic history including divergence, migration events, and population dynamics based on a coalescent simulation‐based method. We used easySFS (https://github.com/isaacovercast/easySFS) to generate the observed SFS and specified a nuclear mutation rate of 3.5 E‐9 per site per generation following the estimates for *Drosophila melanogaster* (Keightley et al., 2009). Eighteen demographic models were tested in three steps (Tables S1–S12). First, we tested the best‐fit demographic model for the topological relationships among three clades with three divergence models (dichotomous model, hybrid speciation model, and simultaneous speciation model). Second, we tested the gene flow among three lineages. Third, we examined the effective population size changes during demographic changes. Every model was run independently 50 times using 100,000 coalescent simulations and 20 optimization cycles to obtain a global maximum likelihood that could be used to evaluate the results. The run with the smallest difference between the MaxObsLhood and MaxEstLhood was chosen as the best run and as the representative of this model. We compared the best run of each model and finally, obtained the optimal model. To generate 95% confidence intervals (CIs) of the parameter estimates, the best‐fit model, a combination of the initial model selection runs and parametric bootstrapping, was used. First, we simulated 100 replicate SFS from the *_maxL.par file for the best‐fit run. We then performed 50 replicate analyses described above for each of the 100 newly simulated SFS files. Finally, we calculated the mean parameter estimates and 95% CIs from the 100 best‐fit bootstrapping replicates. The populations’ historical demographic reconstruction based on ddRAD data was performed in Stairway plot2 (Liu & Fu, 2020) by inferring population size changes over time based on one‐dimensional folded SFS. We generated an SFS for all samples, CE clade and EA clade (to avoid the incompleteness of a single population, WE clade is not used) using easySFS. The nuclear mutation rate of 3.5 E‐9 per site per generation was following the estimates for *Drosophila melanogaster* (Keightley et al., 2009) and a generation time of 0.5 years was adopted for the analysis.

### 2.5 Ecological niche modeling

In order to reconstruct the distribution dynamics of *M. inconspicuus* in historical climate change, the current ecological niche modeling was constructed using the maximum entropy algorithm in Maxent 3.4.1 (Phillips et al., 2006) and then extrapolated to paleoclimatic layers of the Last Interglacial (LIG) periods, Last Glacial Maximum (LGM) period, and mid‐Holocene era (MH). Geographic records of *M. inconspicuus* were collected from the fieldwork, dried specimens (NKUM), and published literature (Wang & Bu, 2020). After spatial thinning (thinning distance = 5 km), 37 distribution records were finally used for niche modeling. We limited our background extent by the points buffers method (study region buffer distance = 0.5°) to avoid the impact of broad geographical background on prediction ability. Initially, an ENM for current conditions was constructed with all environmental variables. Model validation was performed with 50 replicate runs and 25% of the records for model training. Then, we removed one of the high autocorrelation variables (correlation coefficient of |*r*| ≥ 0.7) according to the explanatory power for the Maxent model. Finally, eight environmental variables were used for niche modeling: annual mean temperature (BIO1), min temperature of the coldest month (BIO6), mean temperature of the driest quarter (BIO9), mean temperature of warmest quarter (BIO10), precipitation seasonality (BIO15), precipitation of wettest quarter (BIO16), precipitation of driest quarter (BIO17), and precipitation of warmest quarter (BIO18). The area under the curve (AUC) of the receiver operating characteristic (ROC) plot was used for model evaluation. For the final distribution areas, we applied the minimum training presence as the threshold to obtain the binary distribution map.

### 2.6 Human disturbance analysis

In order to explore whether human activities had an impact on the survival of *M. inconspicuus* during the Holocene, we used four kinds of grid data of land use and human population estimates which might cause important interference to biodiversity (e.g., cropland area, grazing land population counts, and population density, hereafter cropland, grazing, ppc, and ppd) from the HYDE database 3.2 (Goldewijk et al., 2017) to reveal their correlation with the *N*e values from the early Holocene (10,000 BC) to AD 2017. Historical variations of these four categories were extracted based on the location information of populations. In addition, we project human disturbance factors into the geographic map with altitude information to intuitively show the location and degree of these factors. The correlation between cropland area and *Ne* from 7000 years ago to now has been calculated by the Pearson correlation analysis.

## 3 RESULTS

### 3.1 Single nucleotide polymorphism (SNP) calling and mitochondrial data mining

After filtering, the number of reads mapped to each individual ranged from 1,698,402 to 25,279,211, with 6,456,510 reads per individual on average. The final RAD_80 SNP dataset contained 393,789 SNPs with 12.02% missing sites and the USNP_80 dataset contained 28,051 unlinked SNPs.

The mitogenome of *M. inconspicuus* (GenBank accession number: OL944394) was a double‐stranded circular DNA molecule that contained 37 genes, 22 tRNA genes, 13 protein‐coding genes, and 2 rRNA genes. A near‐complete mitogenome of 15,316 bp in length was sequenced without a partial sequence of the control region (Table S2, Figure S2). Based on the strategy of second‐generation data mining, the results of mitochondrial data acquisition were optimistic with only 6.52% missing data concentrated in a few individuals. The mitochondrial data integrity of 108 individuals (84% of all) exceeded 90%, and a mitochondrial matrix was constructed with these samples (PCG‐rad dataset with 10,927 bp) for subsequent data analysis in order to reduce the impact of missing data. The correlation between mitochondrial data integrity and the number of raw reads for each individual was not significant (Figure S3).

### 3.2 Genetic polymorphisms, phylogenetic results, and population genetic structure and differentiation

Based on the ddRAD dataset, phylogenetic results revealed 16 populations were divided into three clades (WE, CE, and EA) which corresponded to the geography (west, central, and east). While the mitochondrial data divided the populations into only two groups (CE and EA), WE (GXJX population) and CE merged into one clade (Figures 1a,b, S4).

The Bayesian cluster analysis showed four genetic clusters (orange, red, pink, and blue) based on the ddRAD dataset (best *K* = 4, Table S3). The main genetic component for WE mainly contained an orange cluster with only a few red genetic components; CE (with a red main cluster) and EA (with a blue main cluster) had likely mixed genetic components at the junction of geographical areas (Figure 1a,c; *K* = 2 & *K* = 3 also shown in Figure S5). Most of the pink cluster was mixed in some populations of CE, but was not dominant in any geographic population (Figure 1c). The result of the discriminant analysis of principal components (DAPC) was consistent with those of the phylogenetic result, showing three clusters of *M. inconspicuus*, while EA and CE were closer (Figure S6). However, no differentiation in genetic structure was revealed based on mitochondrial data (best *K* = 1) (Figure S7, Table S4).

**FIGURE 2:**
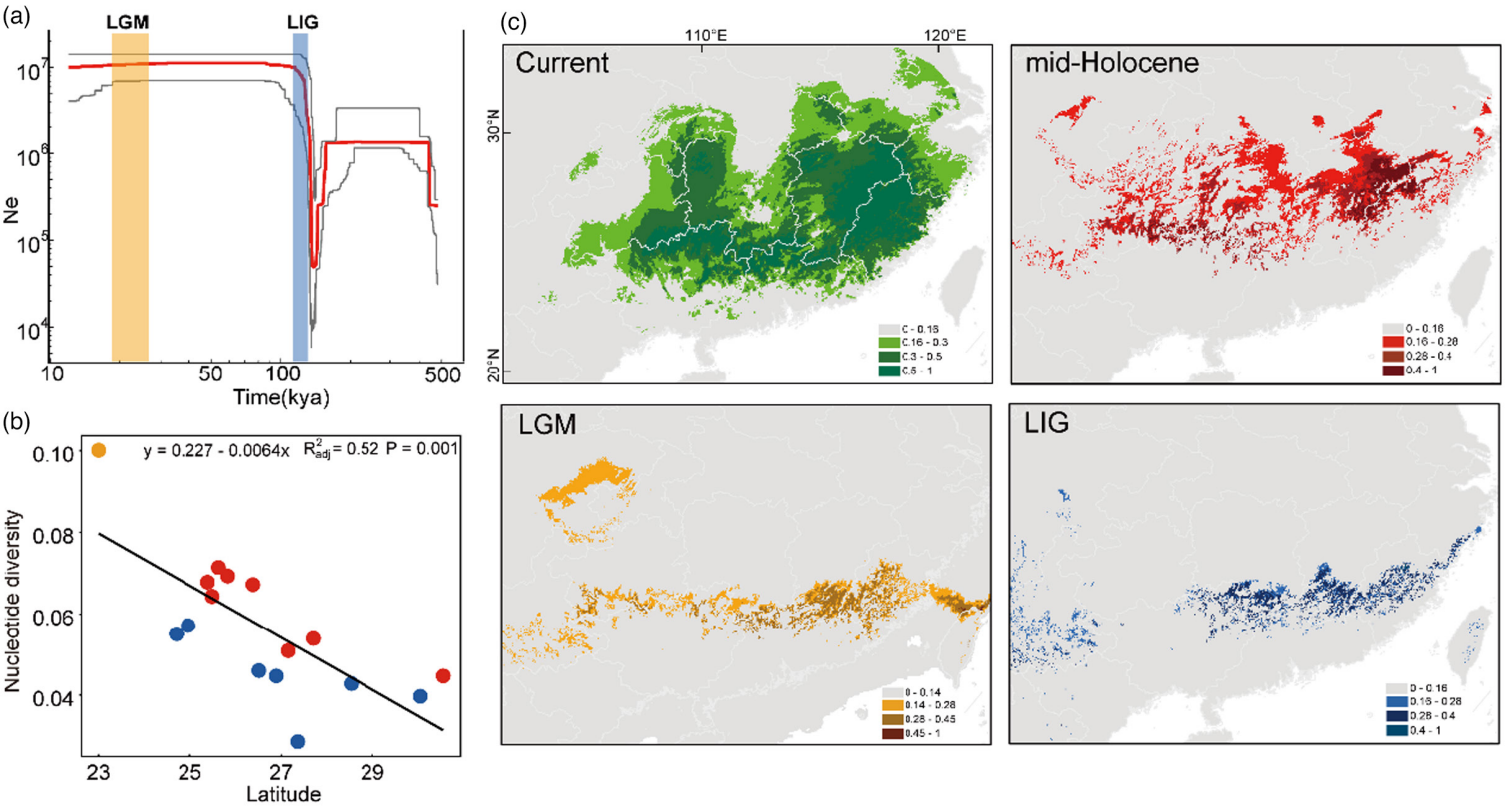
Analysis of population historical evolution dynamics based on ddRAD data of *Malcus inconspicuus*. (a) Historical demographic changes before Holocene (about 12 kya) based on Stairway plot (thick red lines represent); dark gray represents 95% pseudo‐confidence intervals. (b) The correlation between nucleotide diversity and latitude; different colored dots represent three clades (orange—WE, red—CE, and blue—EA). (c) Predicted distribution based on the ENM, different color indicates areas of habitat suitability during different periods (green—current, red—MH, Orange—LGM, and blue—LIG).

The measurements of the indicators of genetic diversity, including *H*_O_, *H*_E_, and *π*, are shown in Table 1. The highest *π* value was found in the GXJX population, and the FJNP population had the lowest *π* value. It is worth noting that the *π* value decreased significantly with increases in latitude (gradually decreasing from south to north) and longitude (gradually decreasing from west to east) (Figures 2b, S8).

Pairwise *F*_ST_ showed the lowest value between the populations within the EA clade (Figure S9). The *F*_ST_ value between the populations of the WE clade and the populations of the other two clades was significantly higher than that found between the other two clades, especially with the populations from the EA clade. However, it is worth noting that the *F*_ST_ value between EA and CE clades was low, similar to that found between populations within the EA clade.

### 3.3 Demographic history model testing and population historical demographic reconstruction

M3_7 was determined as the best‐fit model for the three clades of *M. inconspicuus* (△AIC = 0, Tables S1–S12), as it revealed an initial divergence between EA and the ancestor of WE and CE. Recent gene flow existed among all three clades (the specific value for the recent gene flow is shown in Table S5). A generation time of 0.5 years was adopted to estimate the divergent time. The estimated divergent generations indicated that EA split from the ancestor of WE and CE 0.76 million years ago (mya), and the most recent divergence between WE and CE took place 0.015 mya (Figure 1a, Table S5).

**FIGURE 3:**
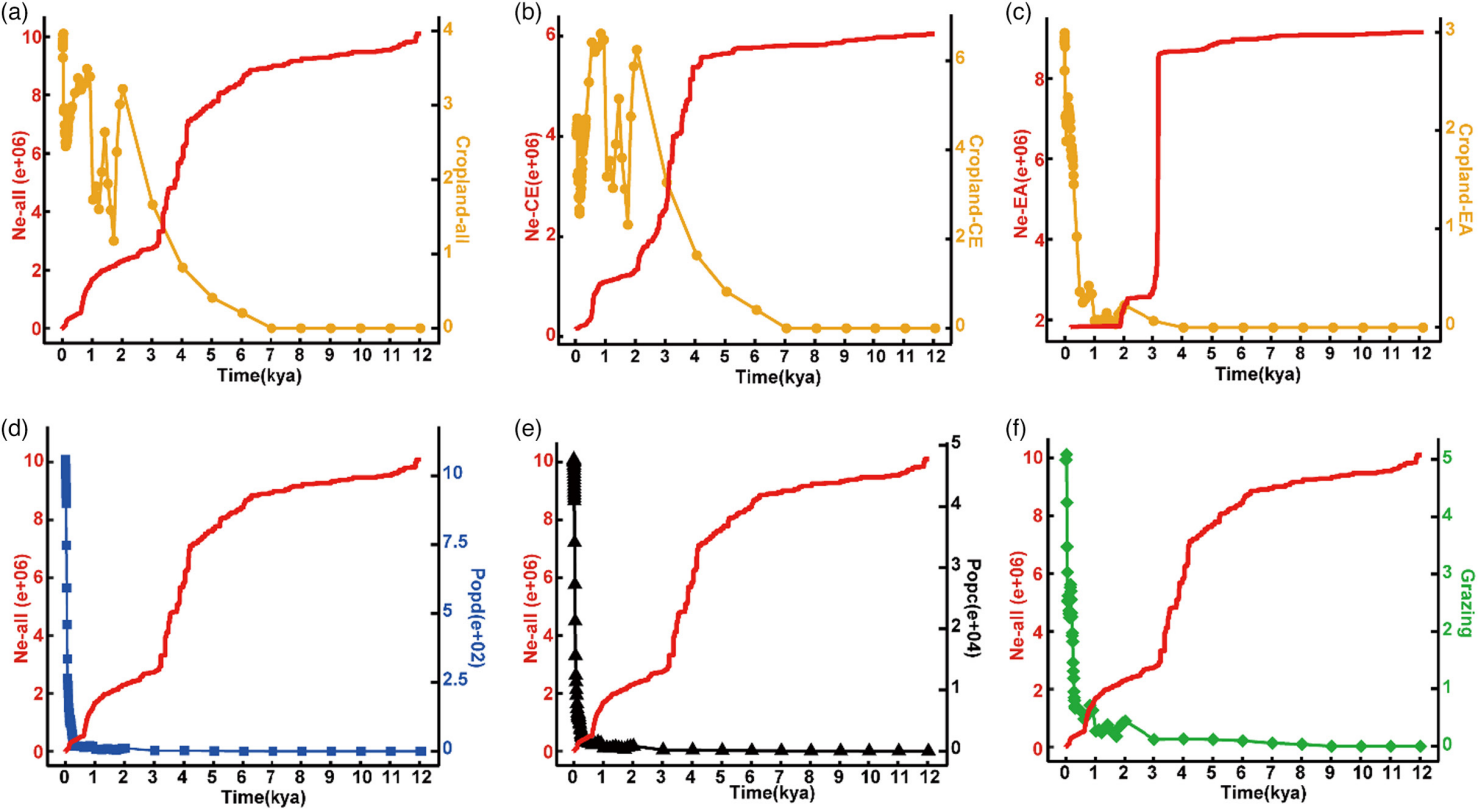
Temporal variations in human disturbance and *Ne*. Values for four categories of human disturbance were extracted based on the populations of distribution. (a) Cropland (total cropland area in km^2^ per grid cell) and *Ne* of all samples. (b) Cropland and *Ne* of CE clade. (c). Cropland and *Ne* of EA clade. (d) ppd (population density per grid cell) and *Ne* of all samples. (e) ppc (population counts per grid cell) and *Ne* of all samples. (f) Grazing (total land used for grazing in km^2^ per grid cell) and *Ne* of all samples.

The stairway plot analysis (Figure 2a) suggested that the Ne of *M. inconspicuus* contracted sharply before the LIG period and then expanded rapidly to the peak. The high level of *N*e remained stable during the LIG and LGM periods until the beginning of the Holocene era (12 kya). After entering the Holocene era, the *N*e showed a gentle downward trend from 7 kya to 5 kya, followed by a sharp decline that started at 5 kya and continued until today (Figure 3). Similar *Ne* change trends were also revealed in CE and EA clades, respectively (Figures S10, S11).

### 3.4 Ecological niche modeling

A relatively high AUC (the area under the curve) value was derived from the current potential distribution of ecological niche modeling (ENM) (AUC = 0.897), indicating the good predictive performance of the model (Figure 2c). The current niche‐predicted distribution was similar to its actual distribution, which is primarily located in the hilly regions of southeast China. When the current niche model was transferred to the LIG period, the potential distribution of *M. inconspicuus* only was located in the southern mountainous area of the current distribution range. The arrival of the glacial age (LGM) caused the suitable area to move southward, to the Nanling Mountains and the southern mountains of the Wuyi Mountains. As the climate became warmer and wetter, the predicted suitable habitat expanded significantly to the north during the MH.

### 3.5 Human disturbance

Within the distribution area of all samples, the cropland area began to grow significantly from 7 kya, accelerated from 5 kya, and reached the first peak at 2 kya, then fluctuated several times from 2 kya to 1.2 kya, and continued to increase to the second peak at about 1 kya, showing a significant negative correlation with the change in *N*e (Figures 3a, S12). However, the increases in grazing, ppc, and ppd showed low correlations with the changes in *N*e, which began to increase from 3 kya and 0.5 kya, respectively (Figure 3d–f). The changing trend of *Ne* and that of human disturbance factors in the CE clade was basically consistent with that of all samples (Figures 3b, S10A). Notably, the EA clade exhibited a slightly different trend (Figures 3c, S10B): within the distribution area of EA clade, although the change curves of ppc, ppd, and grazing showed more drastic fluctuations than those of cropland, only those of cropland increased significantly when the *N*e of the population decreased sharply between 4 kya and 3 kya.

## 4 DISCUSSION

### 4.1 The “multifaceted” effects of mountainous landscape on *M. inconspicuus* in subtropical China

#### 4.1.1 The powerful driver of genetic differentiation

Mountains and waters are generally considered important geographical barriers for the formation of phylogeographical patterns of terrestrial organisms (Liu et al., 2020; Luo et al., 2021; Qu et al., 2015; Smissen et al., 2013). The scattered mountains in the hilly area of subtropical China, one of the major topographic areas in the world, have contributed greatly to population differentiation via the topographic isolation effect (Bai et al., 2014; Liang et al., 2019; Liu et al., 2013; Luo et al., 2021; Tian et al., 2018). In our study, three clades of *M. inconspicuus* (EA, CE, and WE) in subtropical China were revealed based on molecular data, showing consistency with the geographical distribution of several important subtropical mountain ranges in China (Figure 1a): CE and EA clades were mainly located in Nanling Mountains and Wuyi Mountains, respectively, and WE clade was located in the Gulong Mountains. In addition, CE and EA clades were located on both sides of the Luoxiao Mountains, respectively. Therefore, Luoxiao Mountains might be an important geographic barrier for them. These phylogeographical patterns highlighted the significance of mountains in driving the genetic differentiation of *M. inconspicuus*.

The result of demographic history model testing revealed that WE clade had the latest differentiation event with the EA clade. However, the degree of genetic differentiation between the WE clade and the other two clades is significant, even higher than the genetic differentiation between the EA clade and CE clade (Figure S9). Meanwhile, the WE clade has the highest genetic diversity (Table 1). WE clade was located in the Gulong Mountains which are adjacent to the eastern edge of Yunnan–Guizhou Plateau. Compared with the distribution areas of other clades, Gulong Mountains are characterized by a more complex geological environment and less human disturbance (Lu, 2016). The mountainous areas of China are considered to be the cradle of the biodiversity of Hemiptera insects (Li et al., 2021). Natural selection leads to adaptive geographic variation. Therefore, we speculated that, although the WE clade diverged from others at the latest (Figure 1a), the highly heterogeneous geographical environment and the adaptation of the WE clade to local habitats accelerated the formation of high‐level genetic diversity, resulting in its more remarkable genetic differentiation from other clades (Figure S8).

#### 4.1.2 The important refuge to cope with climate fluctuations

During the Quaternary glacial period, although subtropical China was probably non‐ice‐covered during the glaciation, it still underwent severe climate fluctuations, especially dramatically reduced temperature and decreased precipitation (Li et al., 2004; Lu et al., 2013; Zheng et al., 2003).

Montane regions here had relatively mild climates and could provide a sufficient food supply and necessary living environment for phytophagous organisms, making them important refuges for multitudinous species in East Asia (Liu et al., 2012; Luo et al., 2021; Tian et al., 2018). Two divergent events occurred in the historical evolution of *M. inconspicuus*: the ancient divergence that occurred at 0.7 mya and the recent divergence that occurred at 0.015 mya (Figure 1a). These divergent events corresponded directly to the two major glaciations of the Quaternary climatic history: the Naynayxungla and Baiyu glaciations (the Last), which are dated about 0.5–0.72 and 0.01–0.07 mya, respectively (Zheng et al., 2002). These two glaciations are considered to have dramatically influenced the evolutionary history of vertebrates (Qu et al., 2014; Zhao et al., 2013) and insects (Cheng et al., 2021). Therefore, we speculated that the climate fluctuations during the glaciations might have driven different populations of *M. inconspicuus* to further contract their distributions in their respective inhabiting mountains, thereby accelerating the progress of population differentiation. For example, in order to cope with climate change during the LGM, the populations of *M. inconspicuus* transferred to their respective southern mountains to take refuge (ENM showed the southward movement of the suitable area during the LGM, see Figure 2c). In addition, the population historical dynamics revealed a stable population dynamic history of *M. inconspicuus*, which was distributed in Nanling Mountains and Wuyi Mountains during the LGM (Figures 2a, S11), supporting the important protective effects of the hilly regions of Southeast China on organisms during the glaciations.

#### 4.1.3 The diffusion corridor during the postglacial period

Despite the formidable barrier that high mountains can present to biological dispersal, most mountain ranges have relatively low‐altitude passes at irregular intervals that can act as corridors for biological dispersal (Craw et al., 2008). The significant northward expansion of habitats after LGM (Figure 2c) and the obvious decline in nucleotide diversity from south to north along the latitude jointly revealed the postglacial northward diffusion events of CE and EA clades (Figure 2b). Both of the clades spread from south to north along the mountain systems where the two glacial refuges were located. Nanling Mountains consist of five connected northeast–southwest directional mountains that span from west to east, namely Yuechengling, Dupangling, Mengzhuling, Qitianling, and Dayuling Mountains, and connect with the Yunnan Guizhou Plateau, Xuefeng Mountains, and other mountains, providing conditions for the diffusion of biological species (Tian et al., 2015, 2018). Wuyi Mountains had acted as a corridor for southward retreat and northward expansion with its northeast–southwest direction during the Pleistocene (Chen et al., 2012; Tian et al., 2015). Therefore, we believed that the Nanling and Wuyi Mountains provided the northward diffusion corridor for the populations during postglacial period. It is worth noting that, a population hybrid zone of CE and EA clades was formed along Luoxiao Mountains (Figure 1a,c). The contact zone had higher red genetic components in the west of Luoxiao Mountains and higher blue genetic components in the east. Luoxiao Mountains are composed of five northeast–southwest medium mountain ranges (Zhao et al., 2020). We speculated that these mountains provided opportunities for communication between the two clades in the north diffusion process.

However, although the trend of expansion was significant, the northernmost diffusion boundary was still in the subtropical hilly area, indicating that the dependence of species on the environment might have greatly limited their postglacial diffusion range.

### 4.2 The negative impact on *M. inconspicuus* caused by a rapid expansion of cropland in the late Holocene era

The early‐to‐middle Holocene era in many regions worldwide saw the beginning of agricultural economies, placing new evolutionary pressures on terrestrial species (Boivina et al., 2016; Lepczyk et al., 2008; Vargas Soto et al., 2021). With the rapid development of agriculture, large‐scale land reclamation has severely damaged the habitat of many organisms (Basset & Lamarre, 2019; Ntshane & Gambiza, 2016).

**FIGURE 4:**
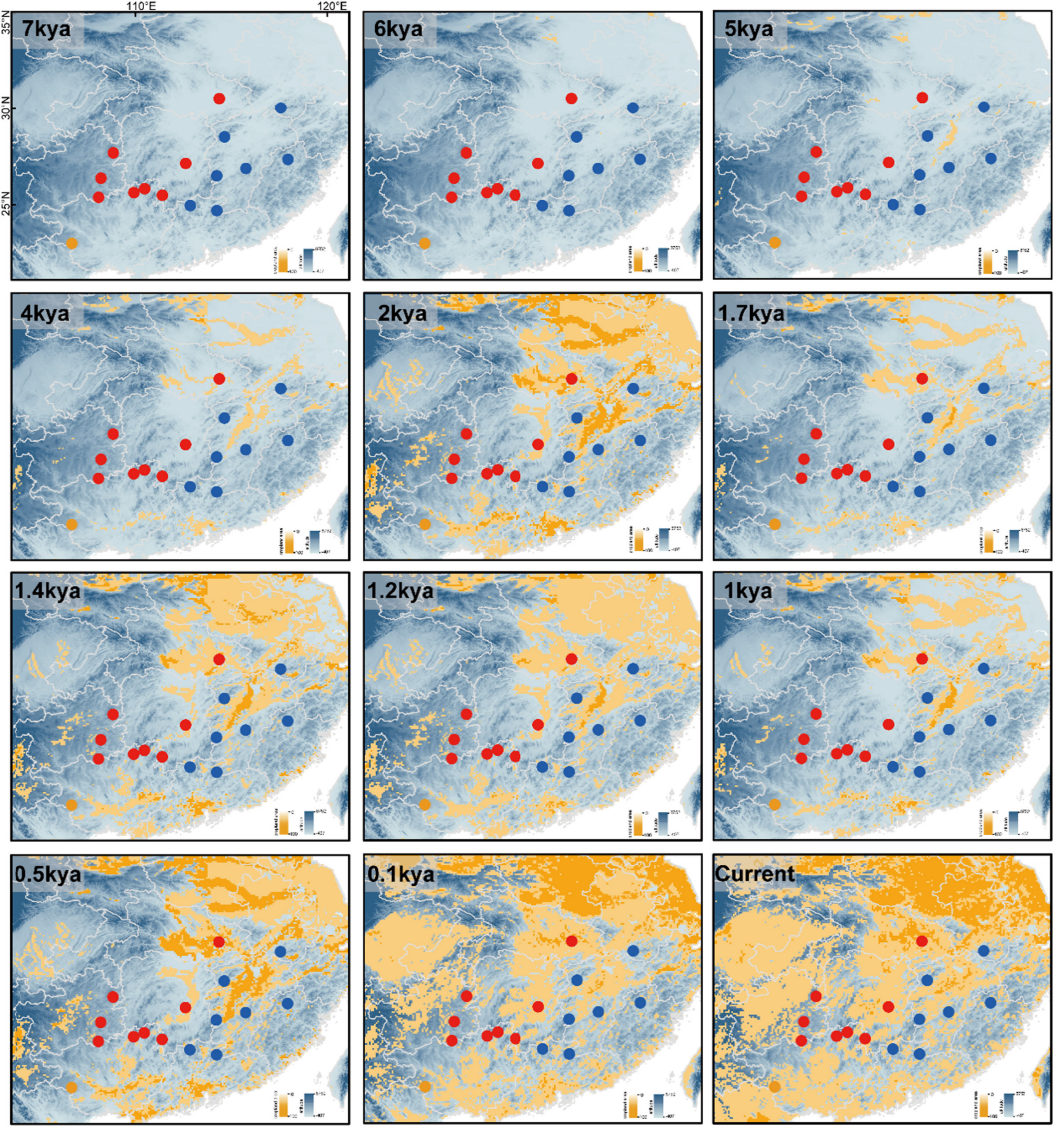
The geographical projection of the cropland area in the subtropical Asia; different colored dots represent three phylogenic clades (orange—WE, red—CE, and blue‐EA).

**FIGURE 5:**
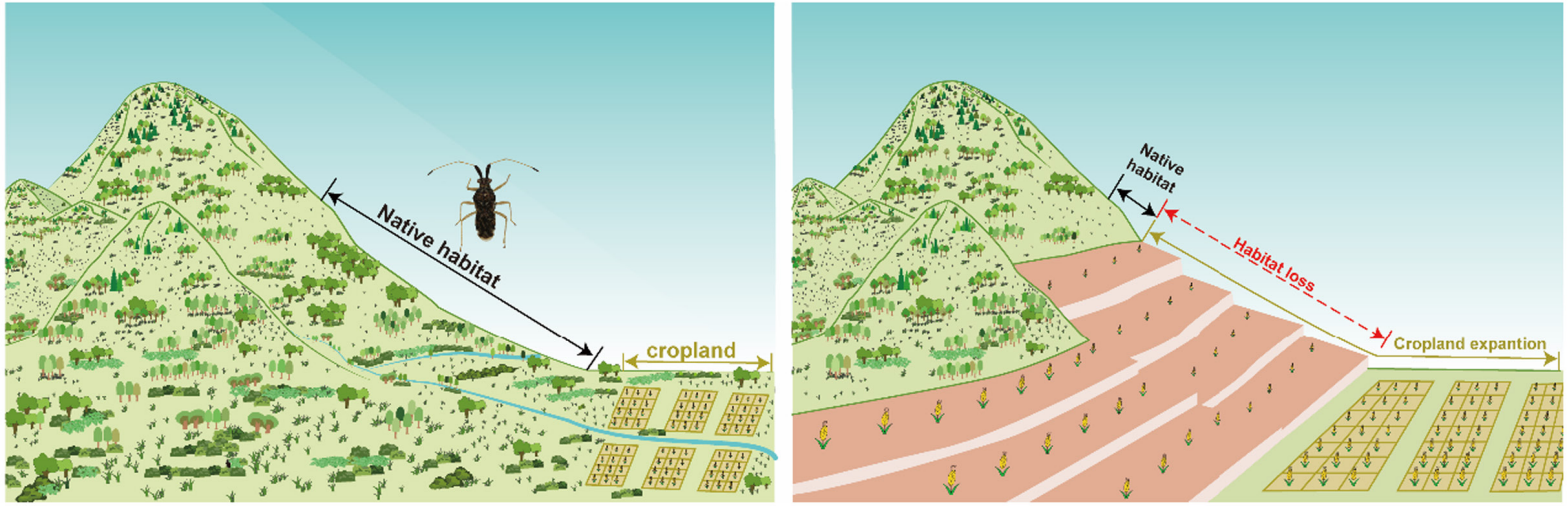
Schematic diagram of habitat loss of *Malcus inconspicuus*.

Our ENM suggests that the distribution range of *M. inconspicuus* populations expanded significantly northward from the middle Holocene era. However, the expansion of distribution has not been accompanied by the increase in population size (Figures 2c, 3). On the contrary, the *Ne* began to decline since about early Holocene (about 7 kya) and decreased sharply from the mid‐Late Holocene (about 5 kya), showing a rapid loss of genetic diversity (Figure 3). At the same time, it showed a significant negative correlation with the change of cropland in the same time and space (Figures 3, S12). We further analyzed the development location of farmland in different periods (Figure 4), and it showed that the development location of cropland was mainly located in plain areas from 7 to 5 kya period. During this period, the *Ne* showed a relatively slow downward trend. However, during the period from 5kya to now, the cropland area expanded into the hilly areas and *Ne* shows a sharp downward trend in this period. *M. inconspicuus* is distributed in subtropical China, and is highly dependent on mountain environment (through evolutionary history and current distribution). Therefore, the destruction of the mountain environment would have a severe impact on it. Based on the above, we speculated that with the development of agriculture in the Holocene era, cropland had gradually expanded into the hilly areas in the subtropical China, resulting in the serious loss of natural habitat and rapid genetic decline in *M. inconspicuus* (Figure 5).

For phytophagous insect species, the change in habitat environment will have a great impact on them (Raven & Wagner, 2021). The blind transformation of the natural environment by human activities has a negative impact both on the diversity and quantity of insects (Carpaneto et al., 2007; Nilsson et al., 2008; Stefanescu et al., 2009; Wagner, 2020). The recent reports and heightened concerns are anchored not only on the loss of species but also extended to the reduction in the number. Such losses could cascade across trophic webs and result in the degradation of ecosystem services (Conrad et al., 2006; Wagner, 2020; Winfree et al., 2015). Therefore, it is very important to monitor and protect the number of species in nature. Although some insect species which are closely related to human agricultural activities (such as pollinators and pests), often showed genetic adaptability to the agricultural environment and recent population expansion events and showed a diffusion route consistent with the change in host planting area (Dorey et al., 2021; Kébé et al., 2017; Lopez‐Uribe et al., 2016; Silva et al., 2020; Vickruck & Richards, 2017; Zhang et al., 2018). However, for species that are highly dependent on natural habitats or have narrow niches, the change in habitat environment by human agricultural activities would bring strong negative impacts and even fatal blows. Therefore, it suggested that we should pay more attention to these species which are highly dependent on the environment, especially in the land development of their distribution areas.

## 5 CONCLUSION

In our study, three clades (WE, CE, and EA) of *M. inconspicuus* have been well supported and revealed the east–west phylogeographical pattern in subtropical China. The phylogeographical pattern consistent with the geographical distribution of several important subtropical mountain ranges (Nanling Mountains, Wuyi Mountains, and Luoxiao Mountains) highlighted the significance of mountains in driving the genetic differentiation of *M. inconspicuus*. Meanwhile, Nanling Mountains and Wuyi Mountains provided an important refuge for *M. inconspicuus* during the LGM period and also provided northward diffusion corridors during post‐glacial period. In addition, Luoxiao Mountains provided a genetic exchange corridor for the EA clade and CE clade in the diffusion process. However, it showed that the northernmost diffusion boundary of *M. inconspicuus* is still in the subtropical hilly areas, indicating that the dependence of species on the environment might have greatly limited their postglacial diffusion range. Significantly, the populations’ sizes of *M. inconspicuus* showed a sharp decline during the Holocene era. In combination with the biological characteristics of species, correlation analysis results, and cropland historical expansion dynamics, we speculated that the rapid expansion of cropland into the hilly areas in subtropical China might be resulting in the serious loss of natural habitat and rapid genetic decline in *M. inconspicuus*. It showed that the species which are highly dependent on natural habitats or have narrow niches might undergo greater impact when the habitat environment was damaged by agricultural activities.

## CONFLICT OF INTEREST

The authors declare that there is no conflict of interest.

## Supporting information


Figure S1–S12.

## Data Availability

Raw RAD sequences: GenBank accession nos. SRR17542071‐SRR17542198. Mitogenome of the newly sequenced *M. inconspicuus*: GenBank accession no. OL944394. PCG‐rad dataset: https://figshare.com/s/f54d315d5221ff286fce. Ecological niche modeling input files: https://figshare.com/s/d1deabd04b953d154daf. Human disturbance analysis input files: https://figshare.com/s/af5eea4f3a106e9cc8b0.
