# Supplementary material for: The anthropogenic effect of land use on population genetics of Malcus inconspicuus
Source: Evol Appl. 2022 Dec 5;16(1):98–110. doi: 10.1111/eva.13512 (PMC9850013; doi:10.1111/eva.13512)
Supplement: Supplementary file 1 — Figure S1–S12. [file EVA-16-98-s001.doc]

**Supplementary Materials for**

**The anthropogenic effect of land use on population genetics of *Malcus inconspicuus***


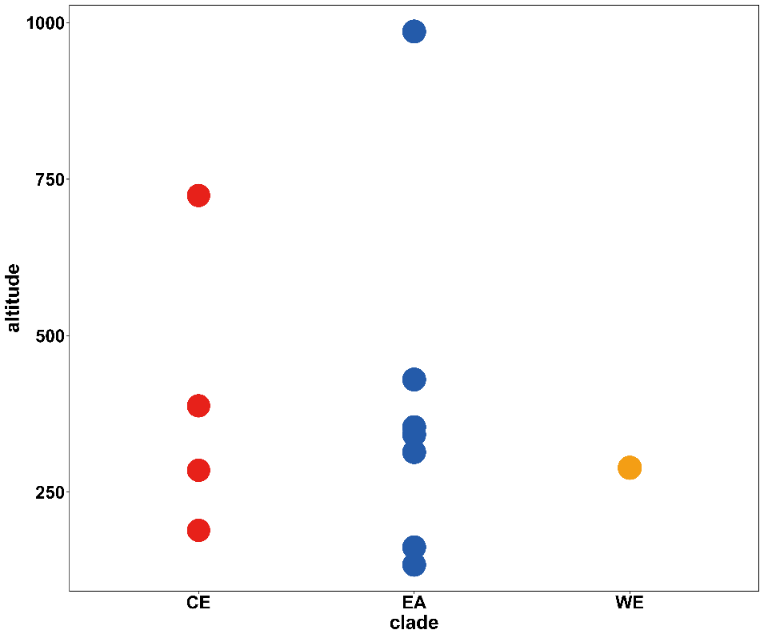


# **FIGURE S1** Altitude of each sample in each clade.


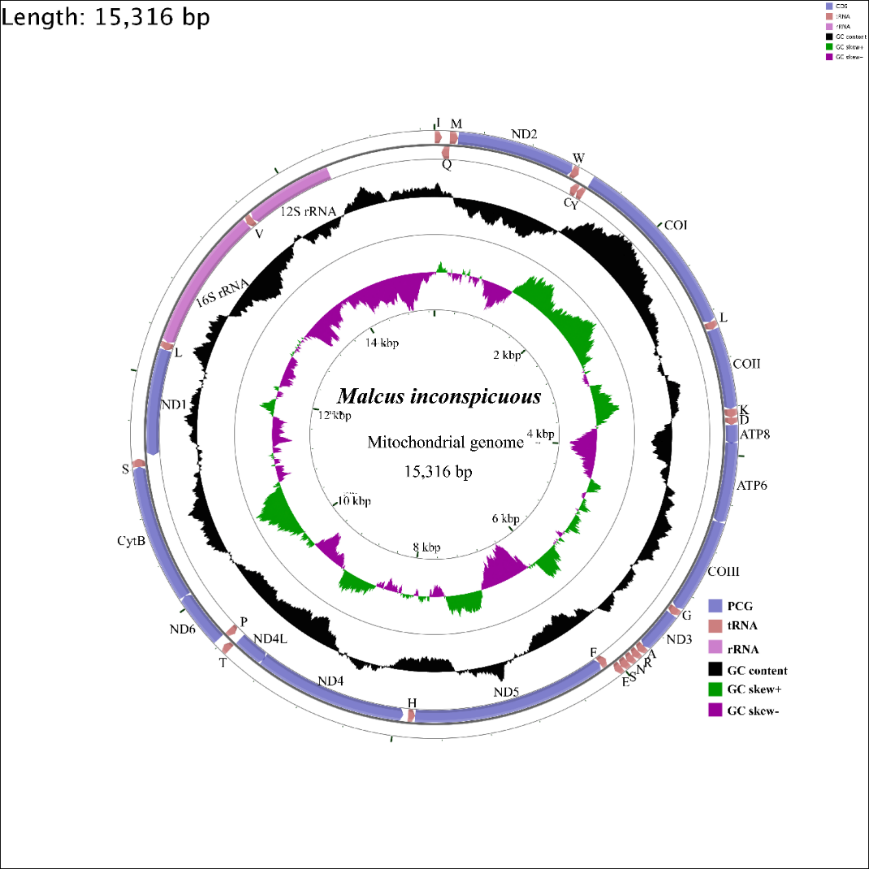


# **FIGURE S2** Mitochondrial genome maps of *M. inconspicuous*. The names of PCGs and rRNAs are indicated by standard abbreviations, while names of tRNAs are represented by a single letter abbreviation. The first circle shows the gene map and arrows indicate the orientation of gene transcription. The second circle shows the GC content, which is plotted as the deviation from the average GC content of the entire sequence. The third circle shows the GC-skew, which is plotted as the deviation from the average GC-skew of the entire sequence. The innermost circle shows the sequence length.


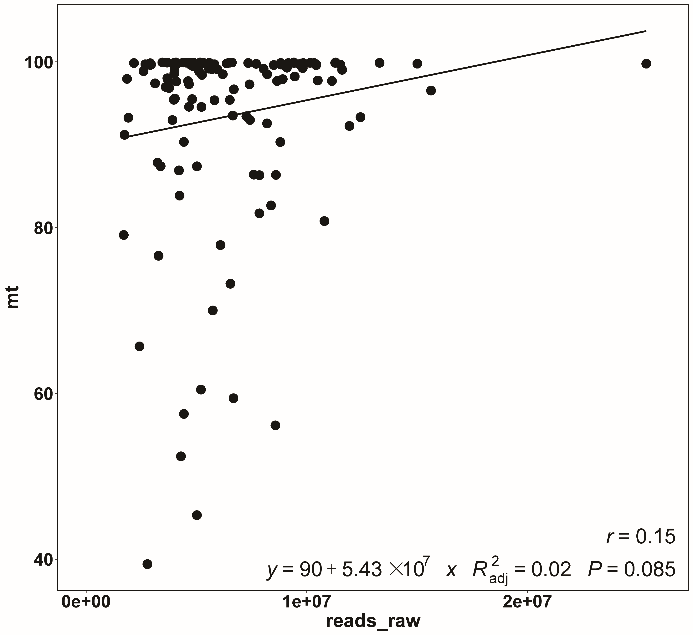


# **FIGURE S3** Correlation between mitochondrial data integrity and the number of raw reads of each individual.


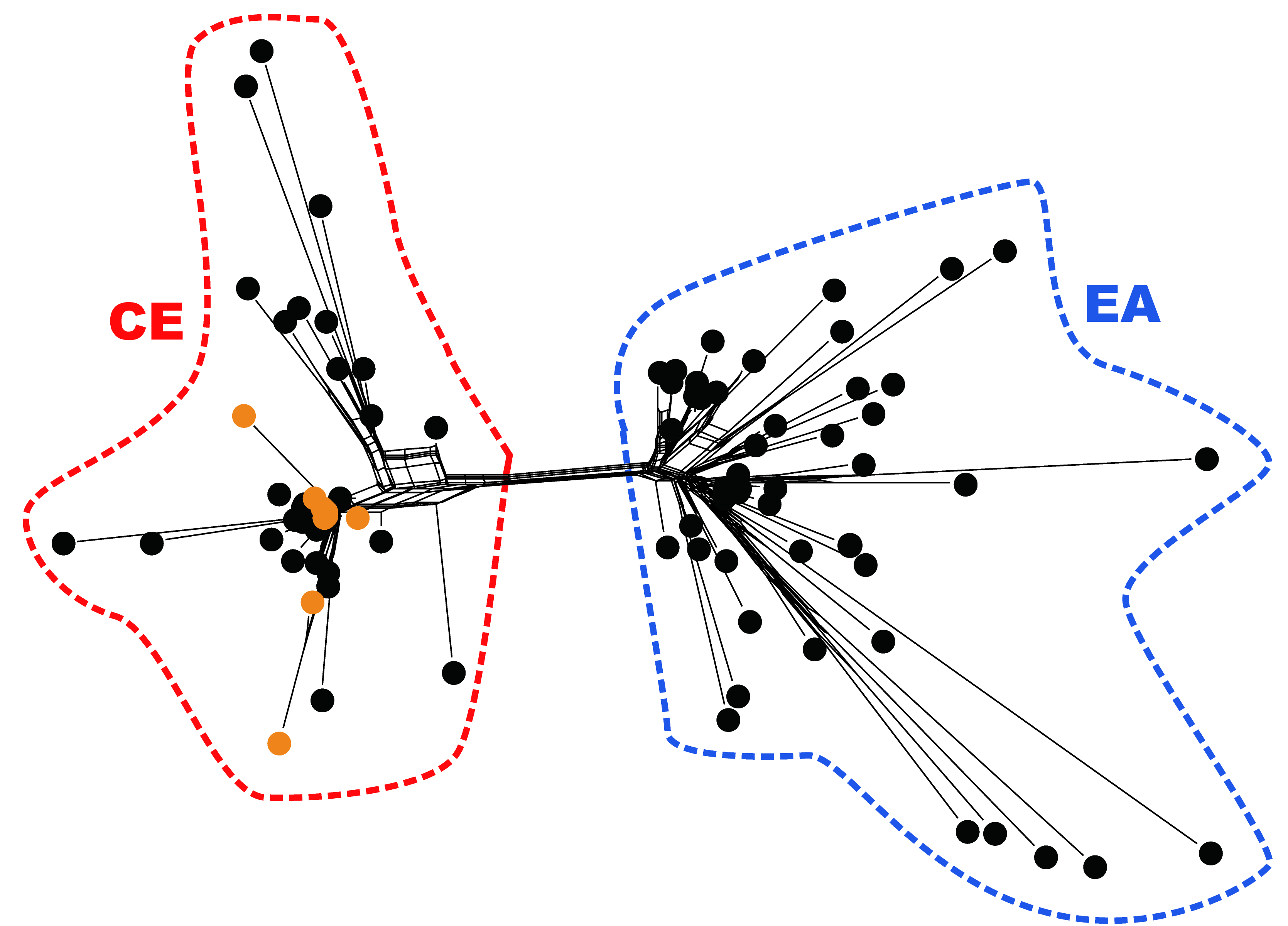


# **FIGURE S4** Phylogenetic network based on mitochondrial data by the neighbor-net method (different colored dotted lines represent two clades: red-CE, blue-EA; orange dots in red-CE represent GXJX population).


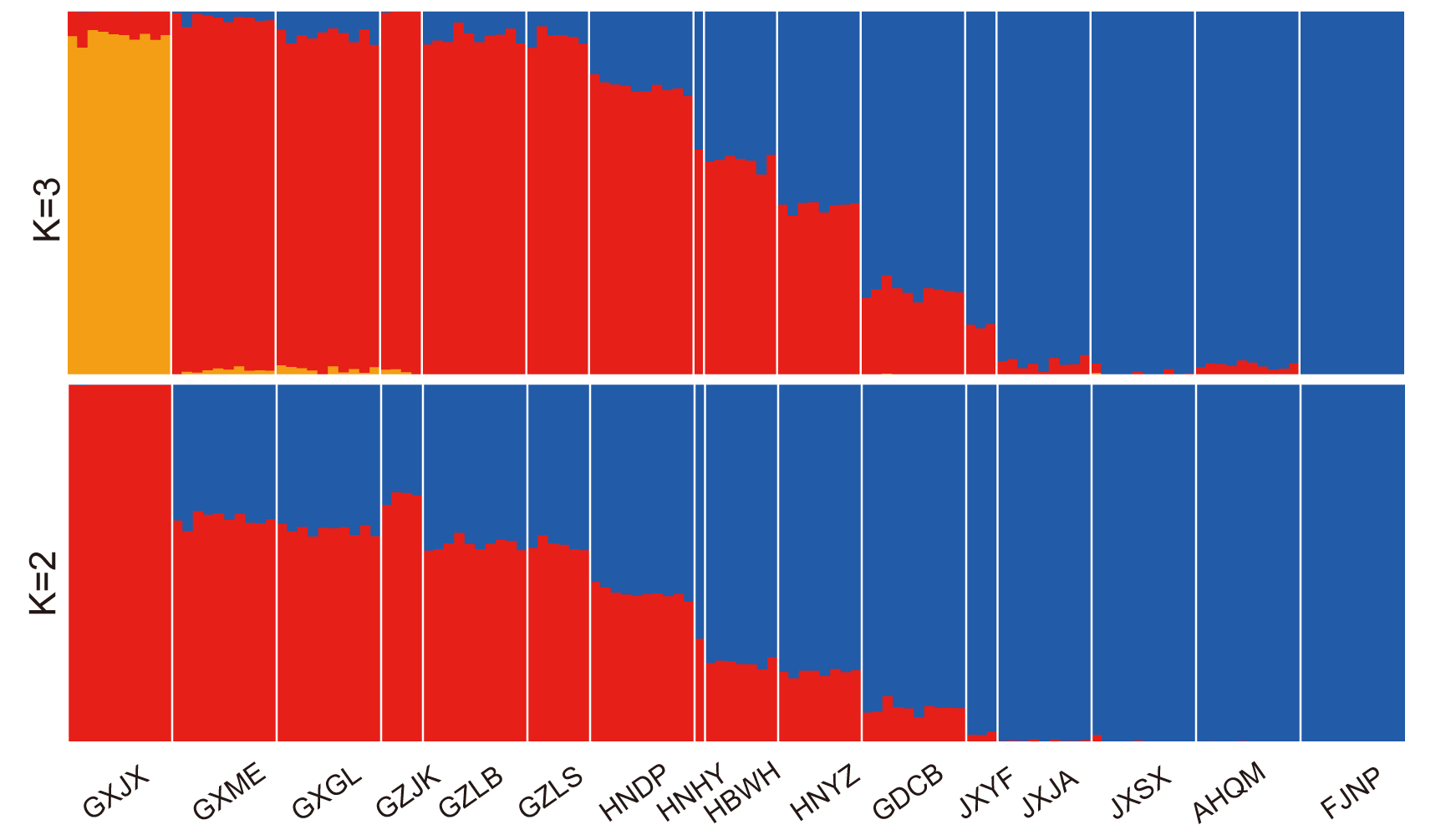


# **FIGURE S5** Structure result with K=2 and K=3.


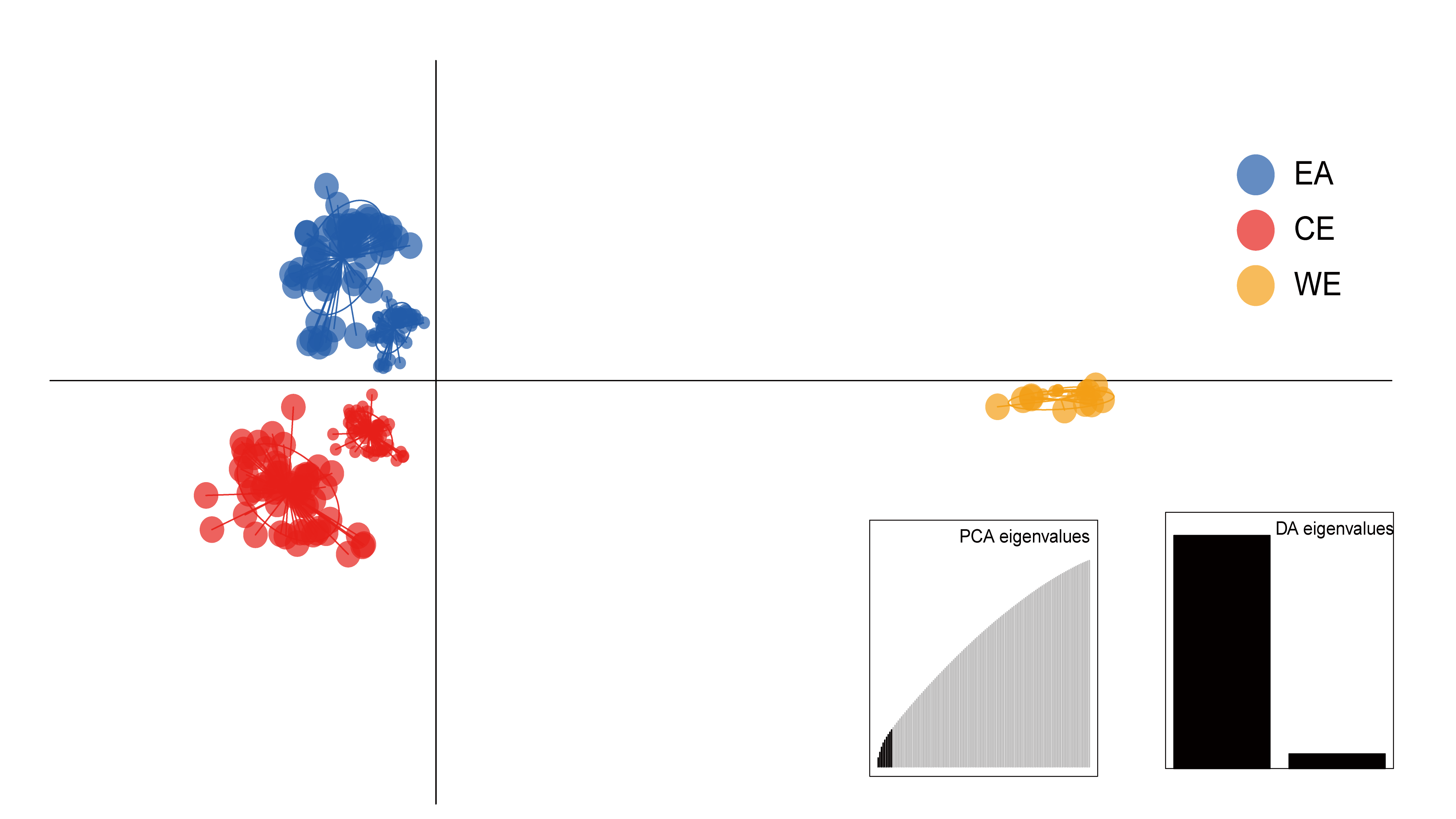


**FIGURE S6** DAPC results based on ddRAD data (different colored dots represent three clades; orange-WE, red-CE, blue-EA). Two axes show the first two principal components of the DAPC.


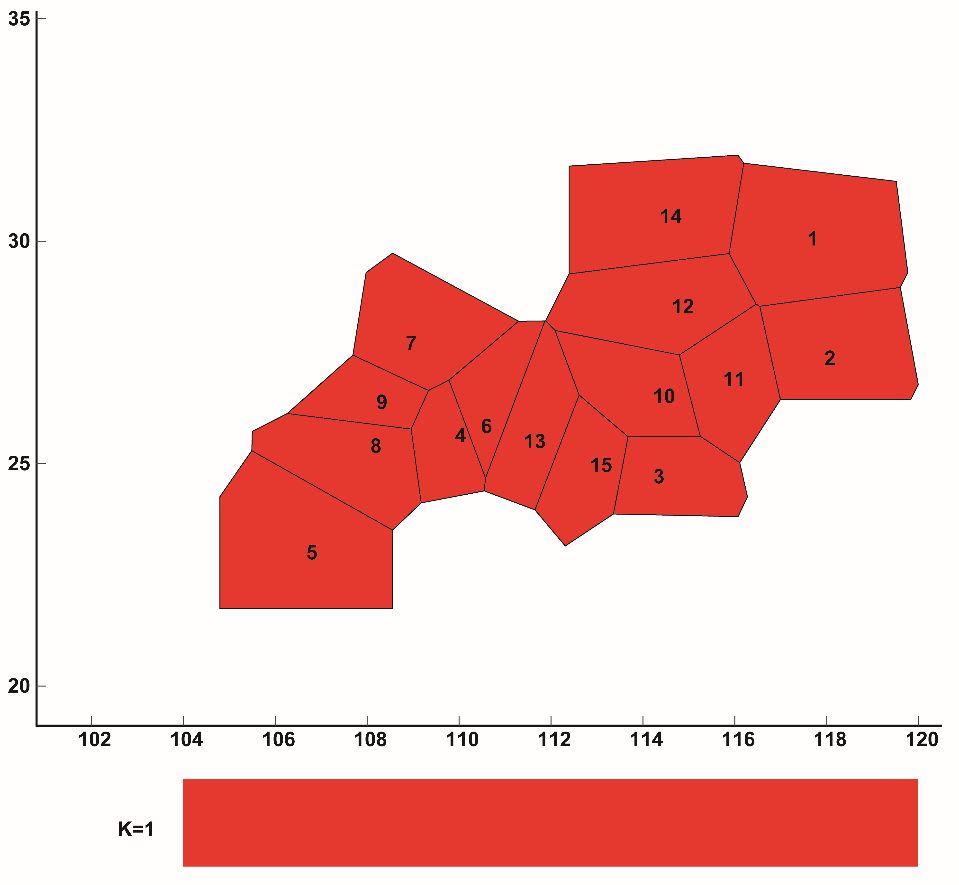


# **FIGURE S7** BAPS result based on mitochondrial data.


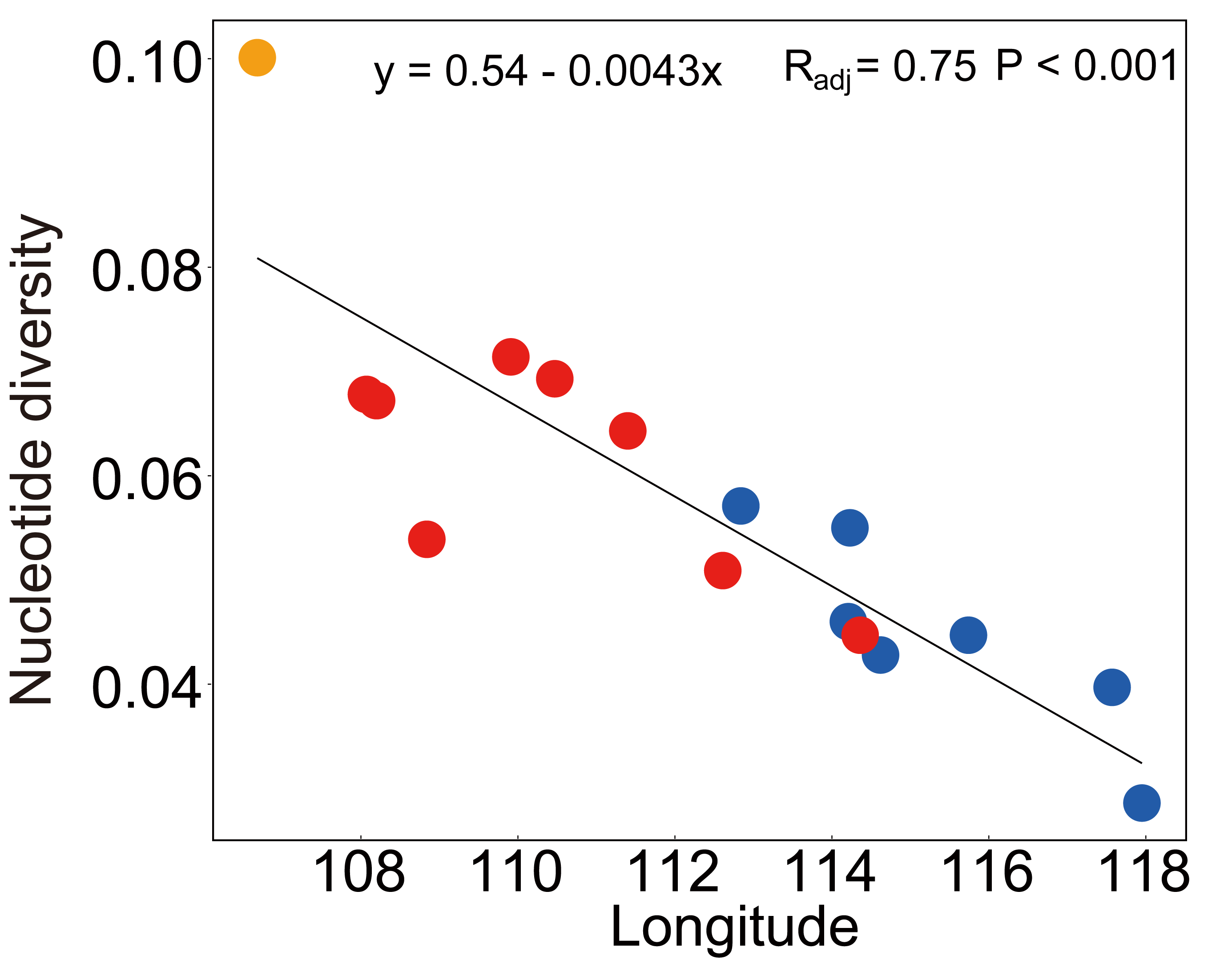


**FIGURE S8** Correlation between Nucleotide diversity and Longitude.


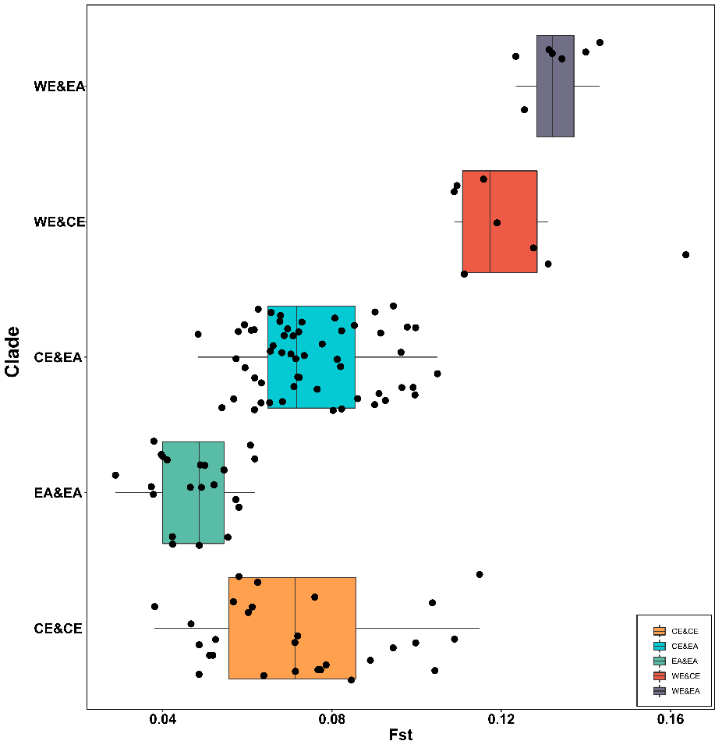


# **FIGURE S9** Pairwise F_ST_ values among different sampling localities


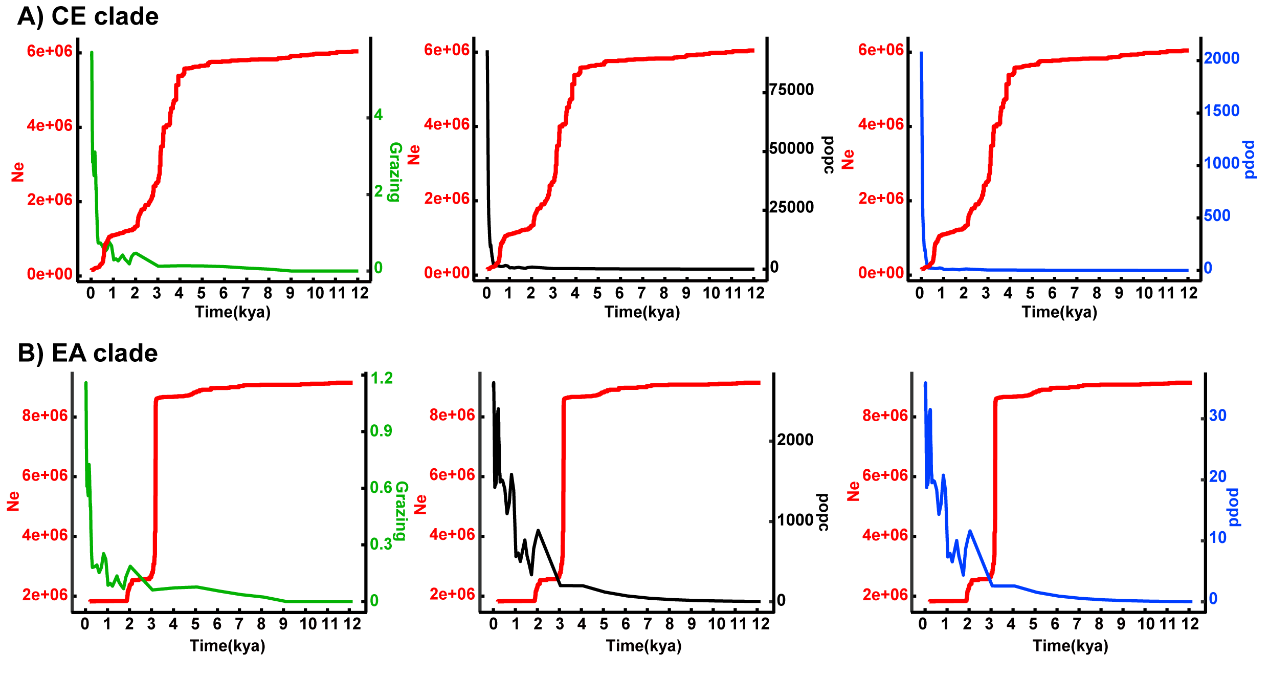


# **FIGURE S10** Temporal variations of Human disturbance factors and effective population size within CE and EA clades.


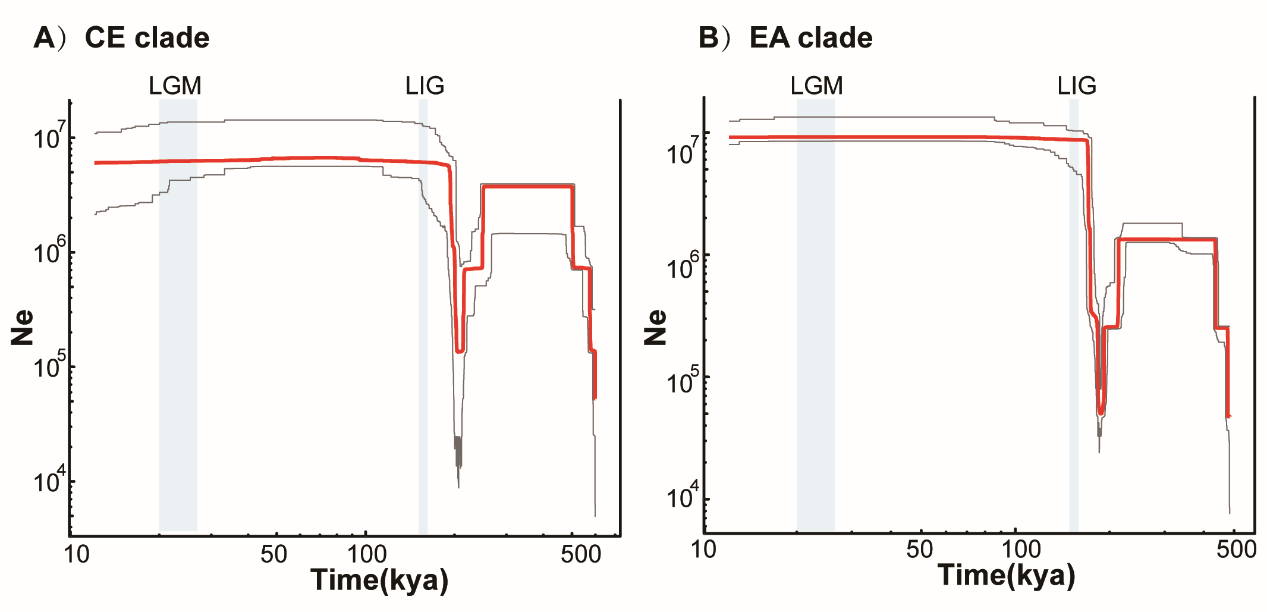


# **FIGURE S11** Historical demographic changes before Holocene (about 12kya) in CE and EA clades based on Stairway plot (thick red lines represent) dark gray represent 95% pseudo-confidence intervals.


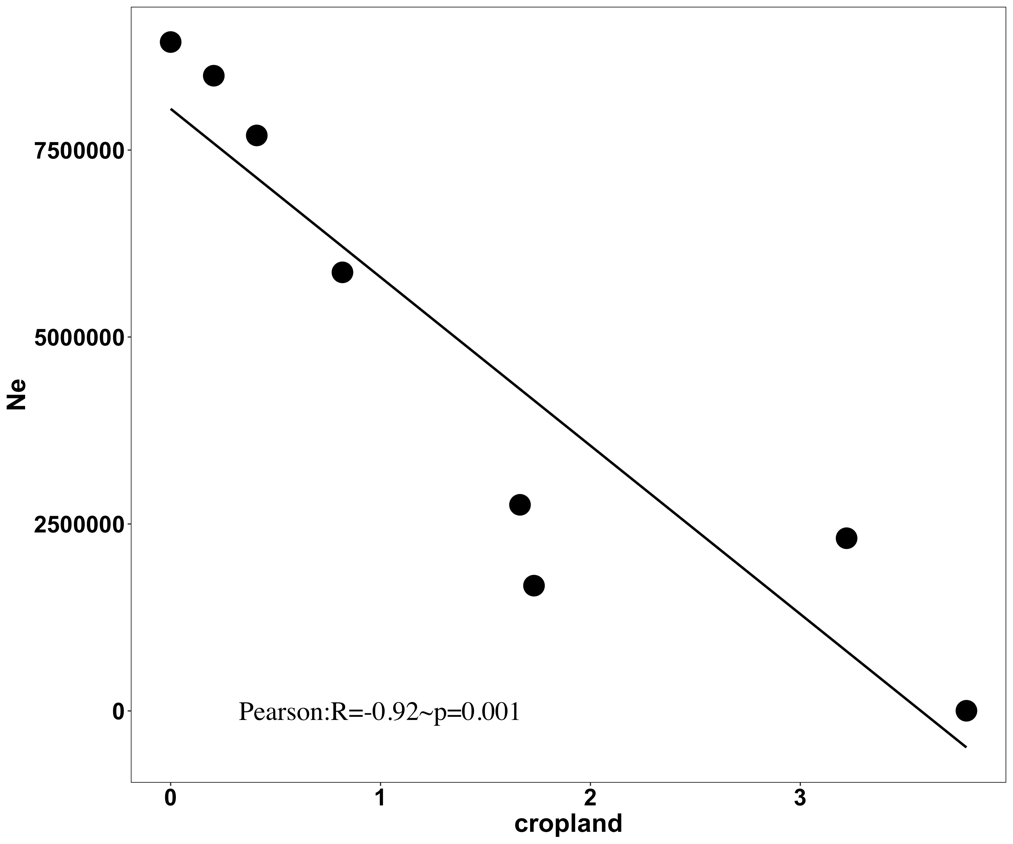


# **FIGURE S12** Pearson correlation results between cropland area and *Ne* from 7000 years ago to now.

# **TABLE S1** Results from the demographic model selection analyses in fastsimcoal2 of *Malcus inconspicuous*.

| **Number** | **Model** | **Description** | **n** | **DeltaL** | **AIC** | **△AIC** |
| --- | --- | --- | --- | --- | --- | --- |
| **Step I：testing the** **best-fit demographic model for the topological relationships** | | | | | | |
| Dichotomous model | | | | | | |
| 1 | M1 | (WE+(EA+CE)) | 5 | 35921.60 | 472549.08 | 62245.59 |
| 2 | M2 | (CE+(EA+WE)) | 5 | 34984.34 | 468232.82 | 57929.33 |
| 3 | M3 | (EA+(WE+CE)) | 5 | 33474.91 | 461279.63 | 50976.14 |
| Hybrid speciation model | | | | | | |
| 4 | M4 | (WE, EA→ CE) | 5 | 35851.53 | 472224.40 | 61920.91 |
| Simultaneous speciation model | | | | | | |
| 5 | M5 | (WE, CE, EA) | 4 | 35744.03 | 471727.36 | 61423.87 |
| **Step II：testing gene flow based on the best-fit model M3 selected in step I** | | | | | | |
| Only with recent gene flow | | | | | | |
| 6 | M3_1 | Symmetrical gene flow between CE and EA | 7 | 28381.23 | 437826.37 | 27522.88 |
| 7 | M3_2 | Asymmetric gene flow from CE to EA | 6 | 29453.93 | 442764.36 | 32460.87 |
| 8 | M3_3 | Asymmetric gene flow from EA to CE | 6 | 32708.52 | 457752.30 | 47448.81 |
| 9 | M3_4 | Symmetrical gene flow between CE and EA, CE and WE | 9 | 24020.60 | 417748.95 | 7445.46 |
| 10 | M3_5 | 1) Symmetrical gene flow between CE and EA. 2) asymmetric gene flow from CE to WE. | 8 | 27261.069 | 432669.8614 | 22366.36807 |
| 11 | M3_6 | 1) Symmetrical gene flow between CE and EA. 2) Asymmetric gene flow from WE to CE. | 8 | 24191.18 | 418532.51 | 8229.02 |
| **12** | **M3_7** | **Symmetrical gene flow among three clades** | **11** | **22402.97** | **410303.49** | **0.00** |
| 13 | M3_8 | 1) Symmetrical gene flow between CE and EA, CE and WE. 2) Asymmetric gene flow from EA to WE | 10 | 24094.46 | 418091.10 | 7787.60 |
| 14 | M3_9 | 1) Symmetrical gene flow between CE and EA, CE and WE 2) Asymmetric gene flow from EA to CE | 10 | 22477.62 | 410645.24 | 341.75 |
| **with recent gene flow and historical gene flow** | | | | | | |
| 15 | M3_10 | Symmetrical recent and historical gene flow among three clades | 13 | 22495.67 | 410734.38 | 430.89 |
| 16 | M3_11 | 1) Symmetrical recent gene flow among three clades 2) Asymmetric historical gene flow from the common ancestors of WE and CE to EA | 12 | 22754.20 | 411922.95 | 1619.46 |
| 17 | M3_12 | 1) Symmetrical recent gene flow among three clades 2) Asymmetric gene flow from EA to the common ancestors of WE and CE | 12 | 22622.63 | 411315.08 | 1011.59 |
| **Step III：testing EPS changed based on the best-fit model M3_7 selected in step II** | | | | | | |
| 18 | M3_7_1 | 1) Symmetrical recent gene flow among the three clades 2) EPS changed when migrants happened | 15 | 22568.59 | 411070.18 | 766.69 |

# **TABLE S2 Organization of the mitochondrial genome of *M. inconspicuous*.**

| **Name** | Position | Strand | Length | Anticodon | Start | Stop | Intergenic |
| --- | --- | --- | --- | --- | --- | --- | --- |
|  |  |  | (bp) |  | codon | codon | nucleotides |
| *tRNA-Ile* | 1-61 | J | 61 | GAT |  |  |  |
| *tRNA-Gln* | 59-127 | N | 69 | TTG |  |  | -2 |
| *tRNA-Met* | 127-195 | J | 69 | CAT |  |  | 0 |
| *ND2* | 196-1173 | J | 978 |  | ATT | TAA | 1 |
| *tRNA-Trp* | 1172-1234 | J | 63 | TCA |  |  | -1 |
| *tRNA-Cys* | 1227-1288 | N | 62 | GCA |  |  | -7 |
| *tRNA-Tyr* | 1289-1350 | N | 62 | GTA |  |  | 1 |
| *COI* | 1352-2885 | J | 1534 |  | TTG | T | 2 |
| *tRNA-Leu(UAA)* | 2886-2950 | J | 65 | TAA |  |  | 1 |
| *COII* | 2951-3620 | J | 670 |  | ATA | T | 1 |
| *tRNA-Lys* | 3621-3690 | J | 70 | CTT |  |  | 1 |
| *tRNA-Asp* | 3691-3750 | J | 60 | GTC |  |  | 1 |
| *ATPase8* | 3751-3903 | J | 153 |  | ATC | TAA | 1 |
| *ATPase6* | 3897-4559 | J | 663 |  | ATG | TAA | -6 |
| *COIII* | 4559-5345 | J | 787 |  | ATG | T | 0 |
| *tRNA-Gly* | 5346-5410 | J | 65 | TCC |  |  | 1 |
| *ND3* | 5411-5758 | J | 348 |  | ATA | TAG | 1 |
| *tRNA-Ala* | 5757-5819 | J | 63 | TGC |  |  | -1 |
| *tRNA-Arg* | 5822-5881 | J | 60 | TCG |  |  | 3 |
| *tRNA-Asn* | 5882-5943 | J | 62 | GTT |  |  | 1 |
| *tRNA-Ser(GCU)* | 5943-6009 | J | 67 | GCT |  |  | 0 |
| *tRNA-Glu* | 6009-6069 | J | 61 | TTC |  |  | 0 |
| *tRNA-Phe* | 6069-6135 | N | 67 | GAA |  |  | 0 |
| *ND5* | 6135-7826 | N | 1692 |  | ATT | TAA | 0 |
| *tRNA-His* | 7827-7887 | N | 61 | GTG |  |  | 1 |
| *ND4* | 7929-9245 | N | 1317 |  | ATG | TAA | 42 |
| *ND4L* | 9239-9511 | N | 273 |  | ATT | TAA | -6 |
| *tRNA-Thr* | 9514-9575 | J | 62 | TGT |  |  | 3 |
| *tRNA-Pro* | 9576-9637 | N | 62 | TGG |  |  | 1 |
| *ND6* | 9640-10089 | J | 450 |  | ATT | TAA | 3 |
| *CytB* | 10089-11222 | J | 1134 |  | ATG | TAA | 0 |
| *tRNA-Ser(UGA)* | 11222-11290 | J | 69 | TGA |  |  | 0 |
| *ND1* | 11308-12228 | N | 921 |  | ATT | TAG | 18 |
| *tRNA-Leu(UAG)* | 12229-12294 | N | 66 | TAG |  |  | 1 |
| 16S rRNA | 12295-13542 | N | 1248 |  |  |  | 1 |
| *tRNA-Val* | 13543-13608 | N | 66 | TAC |  |  | 1 |
| 12S rRNA | 13609-14380 | N | 772 |  |  |  | 1 |

# **TABLE S3** Structure result.

| **K** | **Reps** | **mean est. LnP(K)** | **stdev est. LnP(K)** |
| --- | --- | --- | --- |
| 1 | 10 | -861498.63 | 18.015182 |
| 2 | 10 | -774074.59 | 106.713734 |
| 3 | 10 | -726424.23 | 383.883076 |
| 4 | 10 | -718545.77 | 14150.85178 |
| 5 | 10 | -6676882.65 | 11006910.2 |
| 6 | 10 | -34491518.11 | 38224880.16 |
| 7 | 10 | -87899989.67 | 60371581.21 |
| 8 | 10 | -55537612.61 | 41632738.63 |
| 9 | 10 | -44298268.48 | 18898212.1 |
| 10 | 10 | -78408277.53 | 82787610.71 |
| 11 | 10 | -60050888.6 | 38633501.56 |
| 12 | 10 | -91091668.8 | 83947462.01 |
| 13 | 10 | -72102679.46 | 49548037.34 |
| 14 | 10 | -91740062.86 | 102476908 |
| 15 | 10 | -100202713.9 | 71546488.26 |
| 16 | 10 | -51832840.52 | 28174396.5 |

# **TABLE S4** Log (marginal likelihood) of different K value in BAPS.

| K | log(ml) |
| --- | --- |
| **1** | **-106961.3025** |
| 2 | -114281.7484 |
| 3 | -122876.0389 |
| 4 | -134741.4932 |
| 5 | -147484.2217 |
| 6 | -161197.5194 |
| 7 | -174840.173 |
| 8 | -188796.2046 |
| 9 | -203282.3787 |
| 10 | -217336.5457 |

***TABLE S5*** *Parameter estimates for the best-fit demographic models. Estimate: medium value [95% pseudo-confidence intervals].*

| **Parameter** | **Description** | **Estimate** |
| --- | --- | --- |
| **Divergence times** |  |  |
| **T1** | Divergence time between EA and WE+CE | 765916 [712478, 819354] |
| **T2** | Divergence time WE and CE | 15372 [13906, 16837] |
| **Migration probabilities** | |  |
| **M_EA-WE_** | Migration rates from EA into WE | 2.44E-07 [1.10E-07, 3.78E-07] |
| **M_WE-EA_** | Migration rates from WE into EA | 2.94E-05 [2.55E-05, 3.33E-05] |
| **M_CE-WE_** | Migration rates from CE into WE | 1.10E-04 [9.57E-05, 1.23E-04] |
| **M_WE-CE_** | Migration rates from WE into CE | 2.79E-05 [2.21E-05, 3.36E-05] |
| **M_CE-EA_** | Migration rates from CE into EA | 1.91E-06 [2.55E-07, 3.56E-06] |
| **M_EA-CE_** | Migration rates from EA into CE | 1.54E-06 [1.35E-06, 1.72E-06] |
| **Effective population sizes** | |  |
| **EA-*N*e** | *N*e of EA | 553545.64 [512708.4, 594382.8] |
| **CE-*N*e** | *N*e of CE | 45526.76 [40394.87, 50658.6] |
| **WE-*N*e** | *N*e of WE | 15716.16 [-1658.959, 33091.279] |
